# Sustained Endothelial Expression of HoxA5 *In Vivo* Impairs Pathological Angiogenesis And Tumor Progression

**DOI:** 10.1371/journal.pone.0121720

**Published:** 2015-03-30

**Authors:** Ileana Cuevas, Hans Layman, Lisa Coussens, Nancy Boudreau

**Affiliations:** 1 Department of Surgery, Surgical Research Laboratory, University of California, San Francisco, San Francisco, California, United States of America; 2 Department of Cell & Developmental Biology and Knight Cancer Institute, Oregon Health & Sciences University, Portland, Oregon, United States of America; Ohio State University, UNITED STATES

## Abstract

HoxA5 is expressed in quiescent endothelial cells (EC), but absent in activated angiogenic EC. To examine the efficacy of targeting HoxA5 therapeutically to quell pathologic or tumor angiogenesis, we generated an inducible, transgenic mouse model of sustained HoxA5 expression in ECs. During pathologic angiogenesis, sustained HoxA5 regulates expression several angiogenic effector molecules, notably increased expression of TSP-2 and reduced expression of VEGF, thus leading to inhibition of pathological angiogenesis in tissues. To evaluate if this impressive reduction of vascularization could also impact tumor angiogenesis, HoxA5 mice were bred with a mouse model of *de novo* squamous carcinogenesis, e.g., K14-HPV16 mice. Activation of EC-HoxA5 significantly reduced infiltration by mast cells into neoplastic skin, an early hallmark of progression to dysplasia, reduced angiogenic vasculature, and blunted characteristics of tumor progression. To evaluate HoxA5 as a therapeutic, topical application of a HoxA5 transgene onto early neoplastic skin of K14-HPV16 mice similarly resulted in a significant impairment of angiogenic vasculature and progression to dysplasia to a similar extent as observed with genetic delivery of HoxA5. Together these data indicate that HoxA5 represents a novel molecule for restricting pathological and tumorigenic angiogenesis.

## Introduction

Angiogenesis is a complex, multi-step process that requires temporally and spatially-regulated changes in gene expression by vascular and vascular support cells. While essential for embryonic growth, angiogenesis in adults is generally limited but reactivated during wound healing and tumorigenesis, where it acts to provide nutrients for the growing tumor mass, as well as routes for metastatic dissemination [1]. Although anti-angiogenic therapies targeting vascular endothelial growth factor (VEGF) can increase cancer patient survival, many tumors activate angiogenesis via multiple signalling pathways and mediators, and may become refractory to anti-VEGF therapy. In certain cases, anti-VEGF therapy has been linked to promoting more aggressive, metastatic phenotypes [2]. Thus, additional studies aimed at identifying more comprehensive approaches to manage tumor angiogenesis are warranted.

We have previously reported that the Homeobox (Hox) family of morphoregulatory genes coordinately activate (or suppress) expression of multiple genes driving angiogenesis in complex and dynamic tumor microenvironments. HoxD3 increases expression of matrix-degrading proteinases, and up-regulates integrins αvβ3 and α5β1 [3,4]. HoxA3 also induces angiogenesis via distinct target genes including urokinase-type plasminogen activating receptor (uPAR) and matrix metalloproteinase (MMP)-14, while HoxB3 directs expression of angiogenic guidance molecules including ephrinA1 [5,6]. Moreover, expression of these pro-angiogenic Hox3 genes are up-regulated in the tumor microenvironment, but subsequently suppressed in quiescent vessels [4].

In contrast to the Hox3 genes, HoxA5 and HoxD10 are expressed in resting quiescent vessels, whereas expression is lost in tumor-associated vessels [7]. Forced expression of HoxA5 in activated, cultured ECs down-regulates expression of the pro-angiogenic VEGF receptor 2 (VEGFR2), ephrin-A1, and interleukin (IL)-6 genes, while simultaneously increasing levels of the anti-angiogenic factor Thrombospondin-2 (TSP-2) [8]. Moreover, in cultured EC, HoxA5 also reduces migration and endothelial permeability via stabilization of adherens junctions [9]. Together these findings indicate that HoxA5 may have tremendous therapeutic potential in limiting tumor angiogenesis and stabilizing hyper-permeable tumor vasculature.

To date, *in vivo* analysis of the potential impact of HoxA5 on tumor angiogenesis has not been evaluated as HoxA5 null mice exhibit defective lung morphogenesis [10], and perturbed intestinal maturation [11] resulting in perinatal mortality. Thus, to directly assess the anti-angiogenic potential of HoxA5 in a *de novo* tumor microenvironment *in vivo*, we generated an inducible, transgenic mouse using the TIE-2 promoter to restrict expression of a HoxA5 transgene largely to ECs, where transgene expression was suppressed until doxycycline withdrawal [12]. Using this genetic model, we examined the impact of sustained EC expression of HoxA5 on post-natal vascular development, pathological angiogenesis and *de novo* tumor progression. In addition, we evaluated the therapeutic potential of topically-applied HoxA5 to limit tumor angiogenesis. Together, these results demonstrate that HoxA5 is a novel and potent regulator of tissue angiogenesis, that may also have therapeutic efficacy in limiting tumor angiogenesis thereby impairing *de novo* tumor progression.

## Materials and Methods

### Ethics Statement

All studies were carried out in strict accordance with the recommendations in the Guide for the Care and Use of Laboratory Animals of the National Institutes of Health. The protocols were approved by the Institutional Animal Care and Use Committee of the University of California, San Francisco (Approval numbers AN088604-02 and AN084236-03).

### Animal Husbandry

Mice were housed under conditions conforming to IACUC and University of California Regulations. The *Tie2-tTA* mice were kindly provided by Dr. Rong Wang, University of California, San Francisco [12]. *TRE-HoxA5* mice in an FVB/n background (in which *TRE* designates the tetracycline response element) was created at the UCSF Helen Diller Family Comprehensive Cancer Center, Transgenic Core Facility. Briefly, FVB/n embryos were injected with linearized *TRE-HoxA5* which contained the open reading frame for human HoxA5 cloned under TRE promoter [12]. The injected embryos were then transferred to pseudo-pregnant recipient mice. Presence of the HoxA5 transgene in the progeny was assessed by genomic Southern blot and by PCR genotyping of tail DNA using oligonucleotide primers HoxA5-F (5'aatgagctcttattttgtaaactc-3’) and R (5'-tcagatactcagggacggaaggc-3’) when DNA was successively amplified for 30 cycles at 95°C 60 seconds, 56°C 30 seconds and 72°C 30 seconds. Three founders showed detectable mRNA HoxA5 expression in different tissues. The one with the highest levels of HoxA5 expression was used in most experimental procedures. To suppress HoxA5 expression in embryos and in neonates, doxycycline (Dox) was given to pregnant and lactating females in the diet (Dox pellets, Bio-Serv, Frenchtown, NJ). K14-HPV16 and MMTV-PyMT mice were kindly provided by Dr. Lisa Coussens, Oregon Health Sciences University, and maintained in the FVB/n strain and analyzed as previously described [13]. Four to five animals per cohort were analyzed.

### Isolation of murine lung EC

tTA and HoxA5-tTA mice lungs were minced, collagenase-digested (Worthington Biochemical Corporation, Lakewood, NJ), strained and the resulting cell suspension plated on 10 cm plates coated with 0.1% gelatin (Sigma, St. Louis, MO). Endothelial cells were purified by a single negative (Fc gamma-RII/III antibody, BD Biosciences, San Jose, CA) and two positive (ICAM-2, BD Biosciences) cell sorts using anti-rat IgG-conjugated magnetic beads (Dynabeads, Invitrogen, Carslbad, CA). This procedure has been shown to give >97% pure population [14]. The cells were used between passages 2 to 3. In the studies of Dox regulation in HoxA5 expression, Dox was added to the media at 1μg/ml final concentration. After 48hrs the cells were washed and RNA isolated as described below.

### Gene expression analysis

Total RNA was extracted from lung or skin with TRIzol (Invitrogen) and further purified with RNAeasy columns (Qiagen, Carlsbad, CA) and in column DNase digestion (Qiagen). One microgram of total RNA was reverse transcribed using Moloney Murine Leukemia Virus reverse transcriptase (Invitrogen). Quantitative real-time PCR was carried out in triplicate with a 10–20-fold dilution of first-strand cDNA using taqman probes and primers purchased as Assays On Demand (Applied Biosystems, Foster City, CA) for the following genes: β-glucuronidase (*GusB*—reference gene control), *HsHoxA5*, *MmTSP-2*, *MmEphrinA1*, *MmVEGF-A*, *MmCCL-2 and MmCXCL12*. An ABI Prism SDS 7000 (Applied Biosystems) was used according to the manufacturer’s instructions with the following cycling protocol: one cycle of 50°C, 2 minutes; 95°C, 10 minutes; 40 cycles of 95°C, 15 seconds; 50°C, 1 minute. Data was analyzed with ABI Prism SDS 7000 companion software and Microsoft Excel.

### Miles assay

Evans blue (EB) dye (30 mg/kg in 100 μl PBS; Sigma-Aldrich) was injected into the tail vein of 7- to 8-week-old mice. After 1 min, 30 μl of 5% mustard oil (MO) (Phenyl Isothiocyanate, 98%, Sigma-Aldrich) diluted in mineral oil (MnO) (Sigma-Aldrich) or MnO as a negative control was applied to the dorsal and ventral surfaces of the ear; the application process was repeated 15 minutes later. Anesthesized mice were then cardiac perfused, ears removed, blotted dry and weighed. EB dye was extracted from ears in 1 ml of formamide overnight to 48-hrs at 60°C and measured spectrophotometrically at 610 nm in a Victor^2^ 1420 Multilabel Counter (Perkin Elmer former Wallac, Waltham, MA). Data are expressed as mean ± SEM. Comparisons of the amounts of dye extravasation were evaluated by student’s t- test with *p* values less than 0.05 considered significant.

### Animal wounding model

All mice used were between 7 and 8 weeks of age at the time of wounding. tTA and HoxA5-tTA littermates were anesthetized with 2–3% isofluorane in oxygen at 2 liters/minute. The dorsum of the mouse was shaved, sterilized and a 2.0 cm diameter open wound was excised including the *panniculus carnosus* layer. Animals received buprenorphine as needed for pain. Wound areas were measured every 3 days by planimetry, and subsequent analysis was performed using Adobe Photoshop software. For immunohistochemical analysis, wounds were harvested at the described time points by sacrificing the animal and removing the entire wound area, including a 2mm region outside the edge, as well as the granulation tissue around the wound. For immunohistochemical analysis tissues were fixed in formalin and embedded in paraffin, or frozen in O.C.T. compound until sectioning. Representative images were obtained at the wound edge for evaluation of angiogenesis and granulation tissue formation.

### Subcutaneous injection of tumor cells

Tumors originating in MMTV-PyMT females (70–90 days old) were isolated, and tumor cells extracted. After growing the cells in culture, 1X10^6^ cells re-suspended in 100μl of serum-free RPMI media were injected in the lateral body wall in the axillary region caudal to the foreleg of 6–7 weeks old tTA and HoxA5-tTA females. Mice were fed Dox-free food 4 weeks prior to the s.c. injection and throughout the rest of the experiment. Tumors were measured 3 times weekly and tumor volume was calculated as (x•y^2^)/2 where x = length and y = width. After 32 days tumors were isolated, weighed and photographed using a Pentax Optio 230 digital camera. The tumor was divided and cryo-preserved in OCT for further histological analysis.

### Vascular perfusions and fluorescent angiography

Isofluorane-anesthetized mice were injected with fluorescein-labeled *Lycopersicon esculentum* lectin (100 μl, 2 mg/ml; Vector Laboratories, Burlingame, CA) into the tail vein as described [15]. Three minutes after lectin injection, mice were perfused with 4% paraformaldehyde via the ascending aorta for 2 minutes and non-adherent leukocytes were flushed out. Confocal images were acquired on a Nikon Microscope Eclipse 80i. The whole ear was imaged by confocal microscopy and the binary images analyzed for total vessel area. Qualitative analysis of vascular complexity was evaluated by assessing skeletons and staining intensity. These variables include number of branches, junctions, end points, average branch length, and vessel staining intensity compared to background using Image J software using the Skeletonize 2D plug-in module and intensity module.

### Immunohistochemistry

Immunofluorescent staining (IF) for CD31 was performed on 10–20 μm cryo-frozen tissue sections. Tissue sections were fixed in 4% paraformaldehyde for 30min, washed, blocked, and incubated with primary antibody (rat anti mouse CD31, 1:50 dilution; BD) overnight at 4°C, followed by successive PBS washes and application of secondary antibodies (1:500, goat anti-rat 488, Invitrogen), followed by PBS washing and mounting with mounting media containing Propidium Iodine or DAPI (4’, 6-diamidino-2-phenylindole; Vectashield, Vector Laboratories) and analyzed using a Nikon confocal microscope Eclipse 80i. Staining for Ki67 was performed on 5-micron sections of formalin fixed, paraffin-embedded mouse ears at pre-determined time points. Sections were de-paraffinized, antigens unmasked in citrate buffer (pH = 6.0), blocked in normal goat serum, and incubated overnight at 4°C in primary antibody (rabbit polyclonal Ki-67, 1:150 dilution, AbCam). Sections were then stained in secondary rabbit anti-goat (Vector Labs), followed by PBS washing and color retrieval using DAB chromagen (Dako). Sections were dehydrated, counterstained using hematoxylin, rinsed in xylene, and coverslipped with cytoseal. H&E staining was performed over 5-μm paraffin section using a progressive method.

### Western Blot

Western blots of EC isolated from lungs tTA and HoxA5-tTA were probed for HoxA5. Total protein was isolated from cell lysates, loaded onto a 10% SDS-PAGE gel, transferred onto Immobilon-P membrane and blocked with 5% reduced fat milk in T-TBS. Blots were incubated with rabbit anti-peptide HoxA5 (1:50 dilution, Invitrogen) followed by donkey anti-rabbit-HRP at 1:5000 and detected with the ECL detection system (GE Healthcare, Piscataway, NJ).

### Topical Application of HoxA5 expression plasmids

Twenty-five μg of HoxA5 DNA or pHm6 plasmid DNA was re-suspended in a volume of 25 μl of ddH_2_0 and mixed with an equal volume of 1% methylcellulose prepared in ddH_2_0 as previously described [16]. Fifty μl of this solution was spotted onto bacterial plates and allowed to dry at room temperature for 2 hours. The dehydrated pellets were removed intact from the plates with forceps. The methylcellulose pellet containing the HoxA5 or control pHm6 plasmid was applied weekly on both sides of the left and right ear, respectively.

### Evaluation of neoplastic tissues in HPV16 mice

Generation and characterization of K14-HPV16 mice has been previously described[17]. Briefly, K14-HPV16 transgenic mice represent a well-characterized model of multi-stage epithelial carcinogenesis where human papillomavirus type 16 (HPV16) early region genes are expressed under control of a human keratin 14 (K14) promoter, i.e., K14-HPV16 mice [18,19,20,21,22,23]. By 1-mo of age, K14-HPV16 mice develop hyperproliferative lesions (hyperplasias) throughout skin. Between 3-6-mo, these progress into 100% penetrant focal dysplasias and are predisposed to progress into multiple histologic grades of SCC in ~50% of mice on the FVB/n strain background, 30% of which metastasize to regional lymph nodes. Angiogenic vasculature is first evident in hyperplasias, development of which is linked to infiltration of innate immune cells[20,21,22]. Mild hyperplasia, hyperplasia, and dysplasia were quantified using H&E stained 5-micron, paraffin-embedded tissue sections. The non-muscular side of the ear skin (N = 8 mice) was scanned lengthwise at 20X magnification and each field was marked for mild hyperplasia, hyperplasia, and dysplasia based on increased thickness of epidermal tissues (hyperplasia) and the formation and infiltration of non-native granular tissues (dysplasia). The number of fields for each type of neoplastic lesion was averaged by a blinded observer over the total amount of fields obtained to determine the percent of skin lesion present as previously described [17].

### Statistical Analysis

Statistical significance of differences observed in HoxA5 treated versus tTA or plasmid controls was determined using a Student’s *t* test. The minimal level of significance required was *p* < 0.05.

## Results

### Creation and validation of an inducible, endothelial specific transgenic mouse for HoxA5 expression

**Fig 1:**
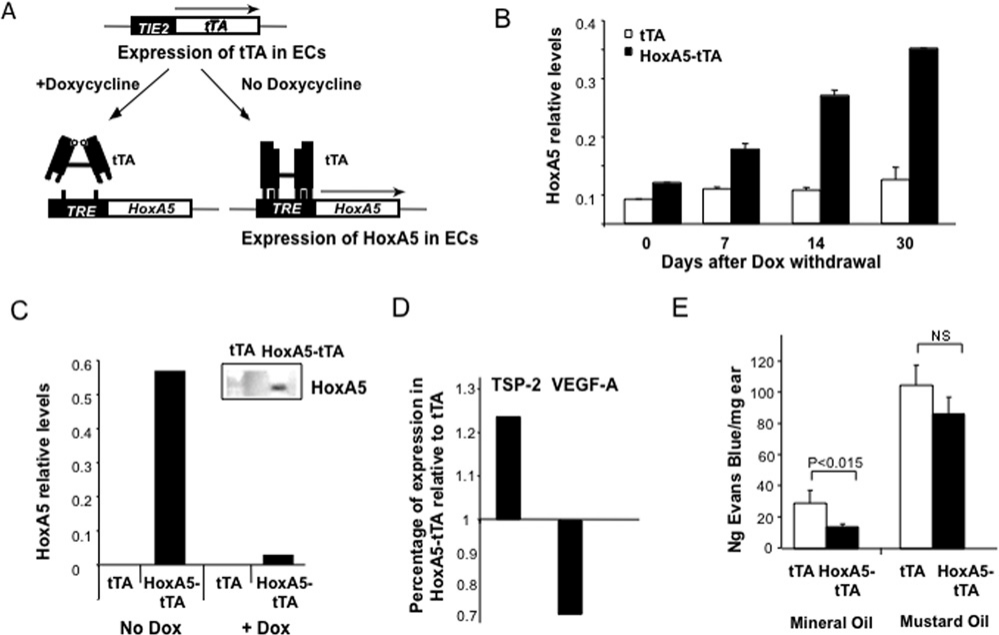
HoxA5 is expressed in EC in TRE-HoxA5/TIE2-tTA mice. (**A**) Schematic of the TRE-HoxA5-TIE2-tTA system for inducible and restricted expression of Hox A5 in ECs in FvBn mice. When the double transgenic mice are maintained on a Dox diet the activator cannot bind the TRE promoter. However in the absence of Dox, tTA binds the TRE activating transcription and HoxA5 expression. Expression of the transgene is restricted to EC by driving tTA using the TIE-2 promoter enhancer [12]. (**B**) Real Time PCR analysis of HoxA5 transgene mRNA expression in mouse liver at various times following withdrawal of Dox from the diet in control TIE-2-tTA (tTA) and TRE-HoxA5-TIE2-tTA (HoxA5-tTA) mice. Results are expressed relative to the housekeeping gene GUSB (n = 3). (**C**) HoxA5 expression levels in ECs isolated from lungs of tTA and HoxA5-tTA mice. Histogram shows HoxA5 mRNA levels measured by real time PCR in the presence of Dox (1μg/ml) or 48 hours following removal of Dox in the cell culture media. Insert shows corresponding Western blot of protein lysates extracted from lung EC isolated from tTA and HoxA5-TA mice and detected via polyclonal antibodies against HoxA5. (**D**) Real time PCR analysis of relative mRNA expression levels of thrombospndin-2 (TSP-2) and VEGF-A levels one month after removal of Dox from the diet of HoxA5-tTA mice. Results are expressed relative to mRNA levels in age-matched tTA control mice lacking the HoxA5 transgene or Dox in the diet. (**E**) Vascular permeability in tTA or HoxA5-tTA mice. Measurement of extravasated Evans Blue dye 30 minutes following topical application of mineral oil (control, left panel) or Mustard oil to induce an acute leakage (right panel; n = 4).

As more than 60% of homozygous HoxA5 null mice die within 4 days of birth, we developed a tissue-specific and inducible genetic model to evaluate the role of HoxA5 in regulating parameters of tissue physiology. Using a bi-genic mouse model, expression of HoxA5 was controlled by a Tetracycline Responsive Element (TRE) (Fig 1A). With this approach, the HoxA5 transgene is suppressed during development and can be selectively activated in angiogenic environments by removing Dox from the diet. To restrict expression of the activating tTA protein to EC, we used the *TIE-2* promoter/enhancer driven tTA system [12]. To suppress HoxA5 expression in the *TRE-HoxA5-Tie2-tTA* (referred as *HoxA5-tTA*) double transgenic mice during development, matings of the parental lines were performed with Dox in the diet. After birth or weaning (3 weeks old), Dox was removed from the diet and within 7 days, HoxA5-tTA mice expressed significantly higher levels of HoxA5 as compared to *tTA* controls (Fig 1B). Expression of HoxA5 continued to increase with time following dox withdrawal with a 4-fold increase in expression, relative to HoxA5-tTA mice at day 0, observed 30 days after activation of the transgene (Fig 1B). To demonstrate that HoxA5 was expressed in EC, we isolated microvascular ECs from lungs of tTA and HoxA5-tTA mice. HoxA5 expression was detected in EC isolated from HoxA5-tTA mice but absent in ECs from tTA mice (Fig 1C). Addition of Dox to growth medium subsequently attenuated expression of HoxA5 in ECs isolated from the HoxA5-tTA mice (Fig 1C). Western blot analysis also confirmed that HoxA5 protein was expressed in EC isolated from HoxA5-tTA mice (Fig 1C, inset). We evaluated expression of HoxA5 in highly vascularized organs, including skin and lung. While the HoxA5 transgene was detected in all tissues analyzed (S1 Fig), expression of the HoxA5 transgene was most abundant in lung, and also highly expressed in skin.

We previously showed that HoxA5 altered expression of angiogenic effector genes, including the anti-angiogenic effector molecule thrombospondin-2 (TSP-2) [8]. Thus we isolated RNA from both skin and lungs, and via real time PCR analysis, confirmed that TSP-2 mRNA in skin increased as a function of HoxA5 expression (Fig 1D) which was accompanied by a 30% reduction in expression of VEGF-A mRNA (Fig 1D).

In addition, we previously reported that HoxA5 also stabilizes adherens junctions and reduces permeability of endothelial monolayers [9]. Thus, to examine the effects of HoxA5 on regulating vascular permeability *in vivo*, we performed a modified Miles assay following topical application of mustard oil in tTA or HoxA5-tTA mice. Under homeostatic conditions, HoxA5-expressing mice exhibited significantly reduced vascular leakage of Evan’s Blue dye as compared to wild type littermates (Fig 1E). However, following application of mustard oil, HoxA5-expressing mice mounted a similar increase in acute vascular leakage as compared to their wild type littermates, indicating that vascular reactivity was not adversely affected by sustained HoxA5 expression.

### HoxA5 and vascular development

**Fig 2:**
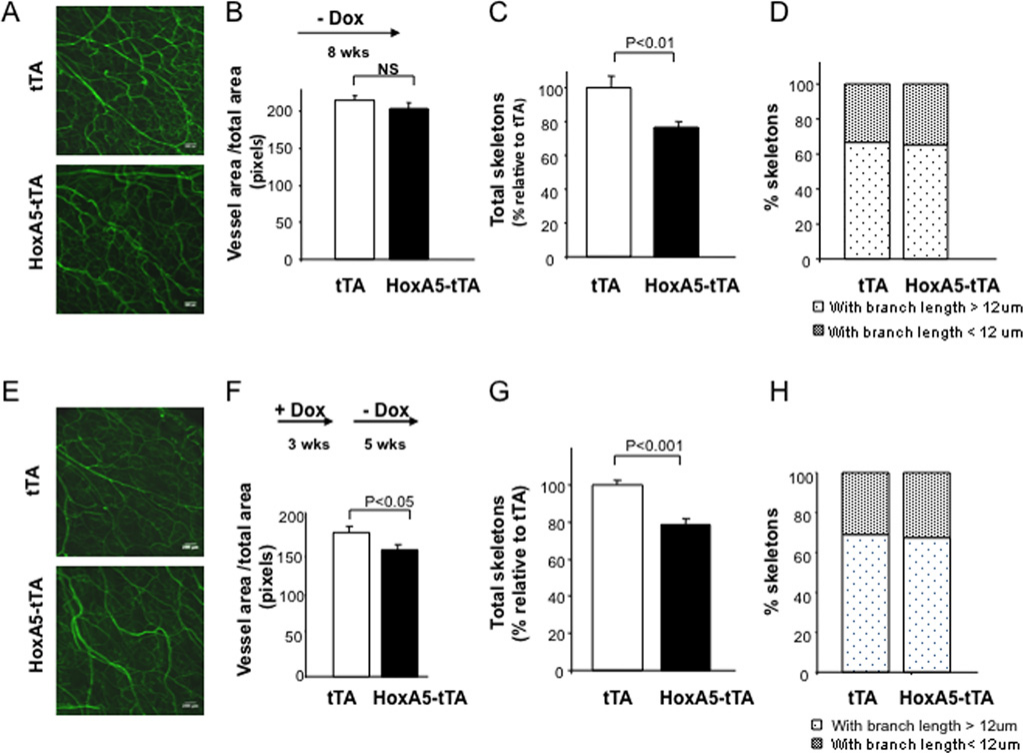
Analysis of ear vessel density in mice. (**A**) Confocal images of FITC- *L*. *esculentum* lectin (green) in whole mounts of ears from tTA (upper) and HoxA5-tTA (lower) mice maintained without Dox (-Dox) during gestation and for an additional 8 weeks after birth. (**B**) Quantitative analysis of vessel area using Image J analysis of confocal images after binary conversion in mice maintained without Dox (- Dox) during gestation and for an additional 8 weeks after birth. (n = 5). (**C**) Qualitative analysis of vasculature described in A and B in tTA and HoxA5-tTa mice following 2-D skeletonization and analysis using Image J (n = 5). (**D**) Analysis of vascular branch length (>12 or <12μm) in tTA (n = 4) and HoxA5-tTA mice (n = 5). (**E)–(H**) Same as (A)-(D), but in 8 week old tTA (n = 4) or HoxA5-tTA mice (n = 3) in which the HoxA5 transgene was inactive during gestation and the first 3 weeks post-natally (+ Dox). Dox withdrawn at this time and the HoxA5 transgene activated for the following 5 weeks (- Dox).

To evaluate the impact of sustained EC expression of HoxA5 on post-natal angiogenesis and development, we examined the vasculature in ears of mice via confocal microscopy following cardiac perfusion with a FITC-conjugated endothelial specific lectin, e.g., *Lycopersicum esculentum*. Total vessel area, number of vascular skeletons and average branch length were quantitatively evaluated. We initially compared 8 week-old HoxA5-tTA mice and tTA mice in which Dox was removed (-Dox) at the beginning of gestation, allowing constitutive HoxA5 transgene expression throughout embryonic development, and maintained for an additional 8 weeks after birth (Fig 2A). Despite sustained EC HoxA5, the HoxA5-tTA mice developed normally, and were fertile. Interestingly, while the mice showed no significant difference in total vascular density as determined by vessel pixel intensity (Fig 2B) quantitative analysis revealed a moderate (<25%) but significant reduction in the complexity of the vascular network as indicated by a reduced number of vascular skeletons (Fig 2C) with no change in the length of vascular branches.

To examine whether the reduced vascular complexity was linked to HoxA5 expression during development, or instead arose during the post-natal period when the transgene remained active, we suppressed the HoxA5 transgene throughout development and for the first 3-weeks after birth (-Dox), followed by induced HoxA5 expression (+Dox) for an additional 5 weeks (Fig 2E–2H). This resulted in a modest, albeit significant reduction in total vessel area in HoxA5-tTA mice compared to control littermates as measured by vessel pixel intensity (Fig 2F). Quantitative analysis again revealed a similar, modest reduction (~20%) in the total number of vascular ‘skeletons’, or complexity, when HoxA5 was activated at 3 weeks post-natally (Fig 2C). However, there were no differences in the average length of the vascular branches (Fig 2D and 2H). and indicates that qualitative changes in vasculature induced by sustained EC HoxA5 likely arise during post-natal vascular growth and/or remodeling, as sustained expression during embryogenesis resulted in no further exacerbation of this phenotype.

### Sustained endothelial HoxA5 impairs pathological angiogenesis and wound healing

**Fig 3:**
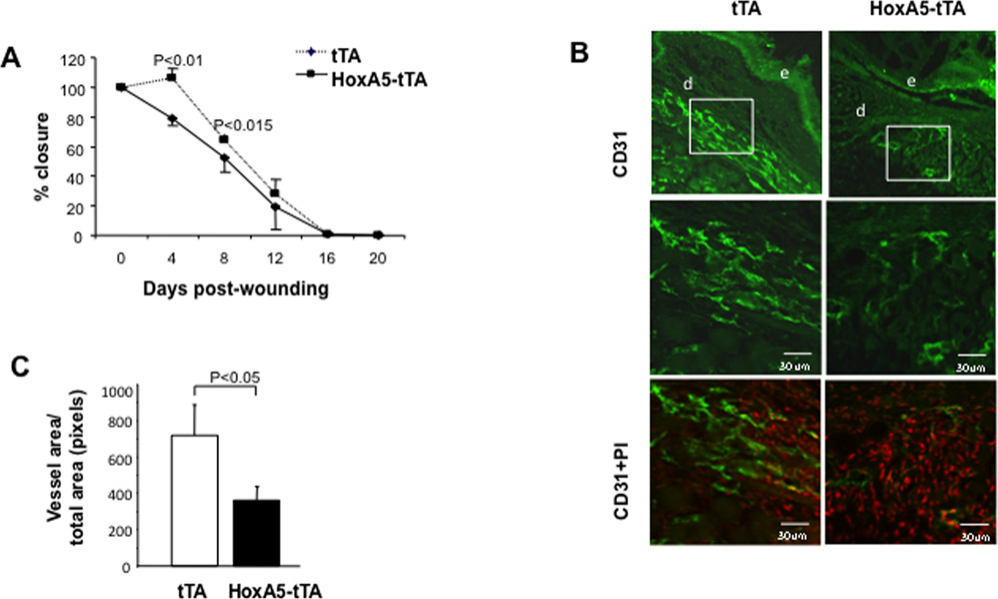
Sustained HoxA5 expression inhibits wound healing. (**A**) Wound closure in tTA (diamonds) and HoxA5-tTA (circles) mice. Wounds were measured every 4 days. HoxA5-tTA mice showed a significant (P<0.01) impairment in closure at day 4 as compared to tTA mice (n = 8). Mice had received Dox for 3 weeks during gestation (+Dox) and then removed for 5 weeks and during the subsequent wound study (-Dox). (**B**) Immunofluorescent analysis of vascular density in wound tissue in tTA (left) or HoxA5-tTA mice (right) 4 days following administration of a 2.0 cm wound and staining with CD31. The upper panel shows lower magnification of positive CD31 staining in the dermis (d) adjacent to the proliferating epidermis (e). The middle panel shows higher magnification of the inset box for positive CD31 staining. The lower panels show corresponding staining with propidium iodide (PI). (**C**) Quantitative analysis of vascular density. Four days following wounding, HoxA5-tTA mice (n = 8) showed a significant reduction (p<0.05) in the number of CD31+ vessels compared to tTA mice (n = 4).

To investigate whether sustained HoxA5 limited pathological angiogenesis, full thickness excisional wounds were administered on dorsal skin of 8 week-old tTA or HoxA5-tTA mice where the transgene was activated 2 weeks prior to wounding (-Dox). Wound healing in HoxA5-tTA mice was significantly reduced at 4 days and 8 days following wounding; times which correspond with peak angiogenic activity during healing of this size wound (Fig 3A) [24]. To quantitate changes in vascular density in the wounds, 2.0 cm wounds were administered and tissue harvested for analysis at 4 days post wounding. The prominent vasculature in tTA mice was observed between the wound edge and the fat tissue (Fig 3B). In contrast to unwounded HoxA5 mice that exhibited a similar vascular density to tTA mice, (Fig 2F) immunofluorescent staining of endothelial CD31 revealed a greater than 40% reduction (*p*<0.05) in the number of vessels at the wound edge from HoxA5-tTA mice as compared to tTA controls (Fig 3C).

### HoxA5 inhibits tumor-induced angiogenesis

**Fig 4:**
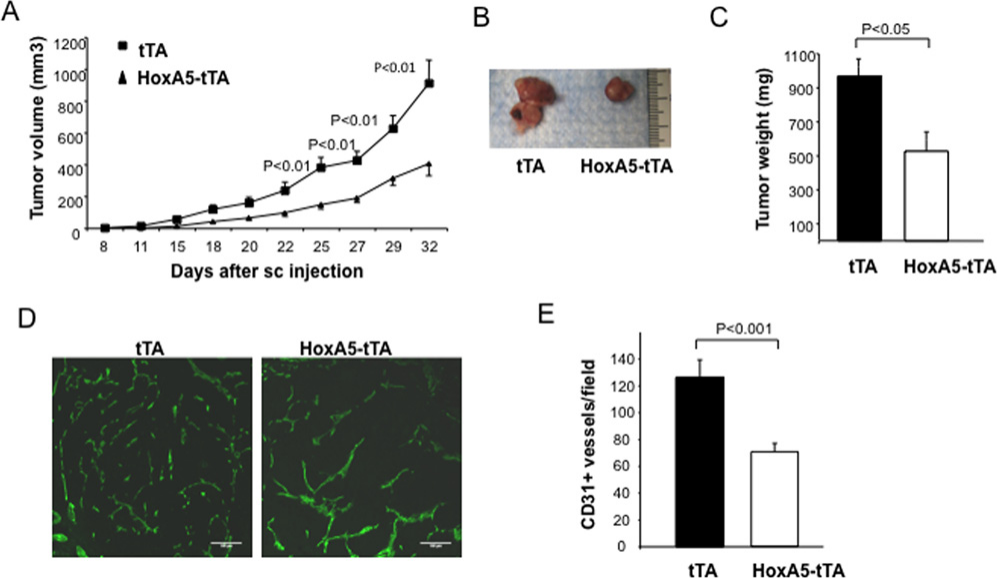
HoxA5 expression in EC inhibits angiogenesis and growth of allograph mammary tumors. (**A**) Tumor volume in tTA (square) and HoxA5-tTA (triangle) 8 week old mice (3 weeks + Dox, 5 weeks—Dox), 32 days following subcutaneous injection of MMTV-PyMT tumor cells into syngeneic female FVB/n mice. The analysis revealed a significant reduction (p<0.01) in volume of tumors grown in HoxA5-tTA mice on days 25 through 32 (n = 5). (**B**) Micrograph showing representative tumors obtained 32 days after implantation of tumor cells into tTA (left) and HoxA5-tTA (right) mice. (**C**) Quantitative analysis of tumor weight 32 days after implantation into tTA or HoxA5-tTA mice. HoxA5-tTA mice showed a significant reduction (p<0.05) in tumor weight as compared to tTA mice (n = 5). (**D**) Immunofluoresence analysis of vascular density in tumors in tTA (left panel) or HoxA5-tTA (right panel). Vascular density was assessed by CD31+ staining of frozen OCT-embedded tissue sections of peri-tumor tissue from each animal. (**E**) Quantitative analysis of CD31+ vessels in tumors isolated at day 32 from HoxA5-tTA mice compared to those from tTA mice. HoxA5-tTA mice showed a significant reduction (P<0.01) in the number of vessels (n = 5).

To examine whether HoxA5 attenuated tumor-induced angiogenesis, control tTA or HoxA5-tTA mice were used to evaluate subcutaneous tumor growth using mammary tumor cells isolated from syngeneic MMTV-PyMT mice [25]. Tumor growth was quantitatively evaluated over a 32 day period revealing a marked reduction in tumor growth in mice expressing EC HoxA5 (Fig 4A). After 32 days, tumor weight in HoxA5 mice was reduced by ~50% (Fig 4B and 4C), and correlated with a significant decrease in tumor vascular density as revealed by quantitative assessment of CD31+ vessels from tumors removed from control versus HoxA5-expressing mice (Fig 4D and 4E).

### Endothelial HoxA5 attenuates the progression of squamous cell carcinoma in K14-HPV16 mice

**Fig 5:**
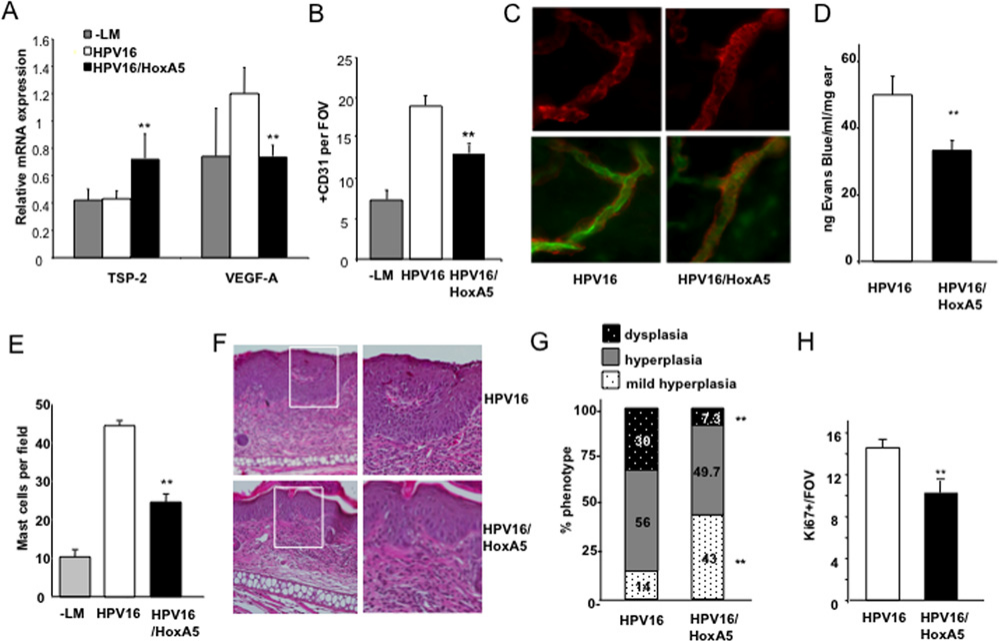
*De novo* neoplastic progression is delayed with sustained expression of HoxA5 in EC. (**A**) Real time PCR analysis of Thrombospondin-2 (TSP-2) and VEGF-A mRNA levels in ear tissue harvested from 5 month old control tTA (-LM), K14-HPV16 (HPV16) or K14-HPV16/HoxA5-tTA (HPV16/HoxA5) mice (n = 5). All mice were given Dox (+Dox) for 3 weeks during gestation, and subsequently removed from Dox (-Dox) for the remainder of the study. (**B**) Vascular density analysis of CD31+ vessels following staining of ear tissue harvested from 5 month old control tTA (-LM), K14-HPV16 (HPV16) or K14-HPV16/HoxA5-tTA (HPV16/HoxA5) mice (p<0.05; n = 5). (**C**) Photomicrographs of confocal images of ear tissues harvested from HPV16 or HPV16/HoxA5 mice. Mice were perfused with endothelial binding FITC-labeled *lycospersicon esculentum* (FITC lectin) and tissues stained for anti-smooth *actin* (SM actin). Upper panel shows images of SM actin coverage of vessels and lower panels show merged images of FITC lectin in ECs and associated SM coverage. (**D**) Vascular permeability in HPV16 or HPV16/HoxA5 mice. Measurement of extravasated Evans blue dye performed 30 minutes following treatment with mineral oil (p<0.05; n = 4). (**E**) Quantitation of mast cell infiltration assessed by toluidine blue staining in ear tissue harvested from 5-month-old control tTA (-LM), K14-HPV16 (HPV16) or K14-HPV16/HoxA5-tTA (HPV16/HoxA5) mice. (**F**) Micrographs showing H&E staining of 5 μm-paraffin sections of 5 month old HPV16 (upper panels) and HPV16/HoxA5 (lower panels) mice. Mice exhibited mild hyperplasia, moderate hyperplasia and dysplasia as measured by epidermal thickness (hyperpasia) or invasion of granulation tissue or immature cell types (dysplasia) in different proportions according to the genotype. (**G**) Quantitative analysis of mild hyperplasia (white), hyperplasia (grey) and dysplasia (black) in 5-month-old HPV16 and HPV16/HoxA5 mice. HPV16/HoxA5 mice exhibited a significantly (p<0.01) higher proportion of mild hyperplasia, and a significantly (** p<0.05) reduced incidence of dysplasia as compared to age-matched HPV16 mice (n = 8). (**H**) Quantitation of Ki-67 positive cells in ear tissue harvested from 5-month-old HPV16 or HPV16/HoxA5 mice where the transgene had been constitutively active since one month of age (n = 3; ** p<0.05).

To further explore the anti-angiogenic potential of HoxA5 during *de novo* tumor progression where early development of angiogenic vasculature accompanies neoplastic progression, we intercrossed HoxA5-tTA mice with a mouse model of *de novo* squamous carcinogenesis, e.g., K14-HPV16 mice [13]. K14-HPV16 mice develop squamous cell carcinomas via a step-wise progression of premalignant lesions (hyperplasia and dysplasia) that are dependent on early development of angiogenic vasculature [18]. In all subsequent experiments, the HoxA5 transgene was activated (- Dox) at one month-of-age, and remained “on” throughout the course of study. Quantitative PCR confirmed that in double transgenic K14-HPV16/HoxA5-tTA (HPV16/HoxA5) mice, HoxA5 expression remained elevated through 5 months-of-age in the absence of Dox, and was expressed at levels comparable to the HoxA5-tTA mice, and significantly higher than in control K14-HPV16-tTA (HPV16) mice lacking the HoxA5 transgene (S2 Fig). Elevated expression of HoxA5 was accompanied by significantly reduced VEGF-A and increased TSP-2 mRNA levels compared to age-matched 5 month-old HPV16 control or negative litter mate (-LM) mice (Fig 5A).

Correlating with altered expression of these angiogenic effector genes, HPV16/HoxA5 mice exhibited reduced vascular density at the 5 month time point as compared to—LM or HPV16 controls, as determined by CD31 quantitation (Fig 5B and S3 Fig). We evaluated neoplastic skin from FITC-labeled *Lycopersicum esculentum* perfused mice, where tissues had been subsequently subjected to immunodetection of alpha smooth muscle actin. Using confocal microscopy we found that expression of HoxA5 resulted in increased smooth muscle (SM) cell coverage of vessels compared to HPV or—LM controls (Fig 5C). Increased SMA has also been correlated with reduced Evan’s blue leakage and this was also reflected by a significant, 38% reduction in vessel permeability (Fig 5D). Tumor angiogenesis in K14-HPV16 mice is dependent on an early influx of mast cells [18] thus, we also stained neoplastic skin sections with toluidine blue to identify infiltrating mast cells, and observed a significant reduction in neoplastic tissue and infiltrating mast cells in HPV16/HoxA5 mice (S3 Fig and Fig 5E) as compared to age-matched HPV16 controls.

We then evaluated age-matched skin from 4 or 5 month old HPV16 and HPV16/HoxA5 mice for the presence of hyperplastic and/or dysplastic skin (Fig 5F). Ear tissue from both HPV16 and HPV16/HoxA5 animals exhibited mild hyperplasia, hyperplasia and mild focal dysplasia. However, the frequency of skin lesions progressing from mild hyperplasia to focal dysplasia in HPV16/HoxA5 mice was significantly reduced as compared to HPV16 mice at both 4 (S3 Fig) and 5 month time points (Fig 5G). Reduced progression to dysplasia in 5 month old HPV16/HoxA5 mice was also accompanied by reduced keratinocyte proliferation as revealed by Ki-67 positivity (Fig 5H). Thus activation of EC HoxA5 in HPV16/HoxA5 mice was reflected by reduced angiogenesis and progression to dysplasia.

### Topical gene transfer of HoxA5 also attenuates angiogenesis and tumor progression in K14-HPV16 mice

To further explore the translational potential of HoxA5 in limiting tumor angiogenesis and/or neoplastic progression, we investigated the effects of a topically-applied HoxA5 transgene in K14-HPV16 mice. For these studies, a methylcellulose-based transgene delivery method, previously developed in our laboratory [16] was used to deliver HoxA5 expression plasmids to neoplastic skin of K14-HPV16 mice. We previously reported that this method efficiently delivers Hox transgenes to poorly-healing cutaneous wounds, and is an effective short-term approach with gene expression lasting 7–10 days and uses a plasmid rather than viral-based expression vector to prevent permanent integration into the host genome [16]. Our previous studies also demonstrated that the transgene remains localized to the site of application, and we confirmed that elevated HoxA5 mRNA in treated ears of K14-HPV16 mice remained local, and was not detected in distal tissues, organs or peripheral blood (S4 Fig).

**Fig 6:**
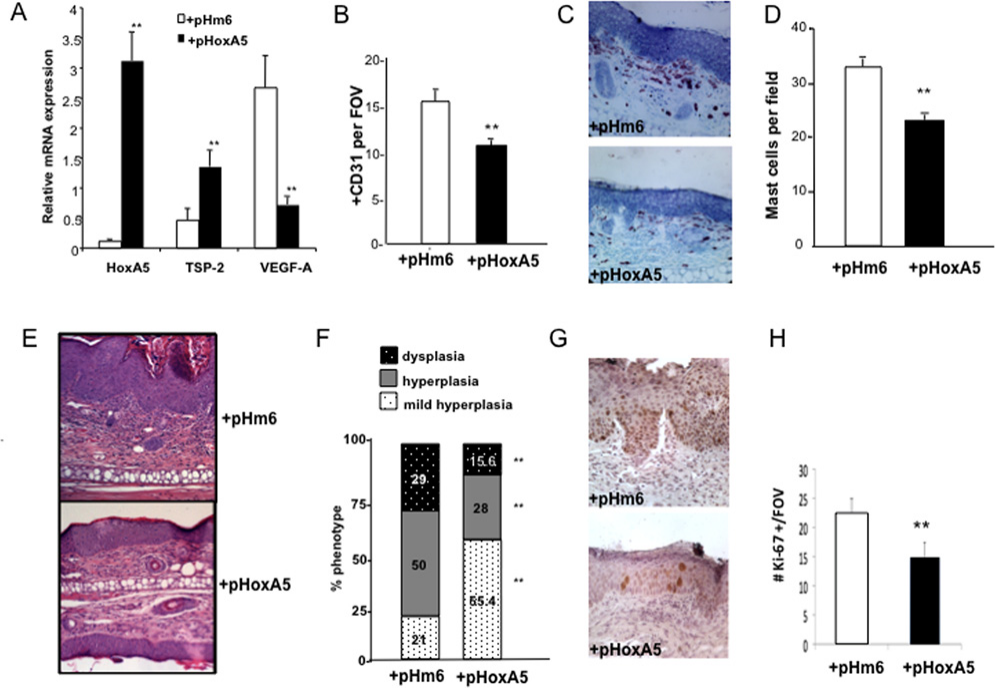
Topical application of HoxA5 expression plasmids inhibits tumor angiogenesis and impairs tumor progression. **(A)** Real time PCR analysis of gene expression in ear tissue from 5 month old K14-HPV16 mice treated with control (+pHm6) or HoxA5 (+pHoxA5) expression plasmids weekly beginning at one month-of-age. Relative levels of HoxA5, TSP-2 and VEGF are expressed relative to the GUSB housekeeping gene (n = 4). (**B**) Vascular density assessed by immunodetection with anti-CD31 antibodies in ear tissue harvested from 5-month-old K14-HPV16 mice treated with control (+pHm6) or HoxA5 (+pHoxA5) expression plasmids for 4 months (**p<0.05; n = 3). (**C**) Toluidine Blue staining of mast cells in ear skin from 5-month-old K14-HPV16 mice treated with control (+pHm6) or HoxA5 (+pHoxA5) expression plasmids for 4 months. (**D**) Quantitation of mast cell influx in ear tissue harvested from 5-month-old K14-HPV16 mice treated with control (+pHm6) or HoxA5 (+pHoxA5) expression plasmids for 4 months. (**p<0.05; n = 3). (**E**) Photomicrographs of H&E stained ear tissue taken from 5-month-old K14-HPV16 mice treated with control (HPV16+pHm6) or HoxA5 (HPV16+pHoxA5) expression plasmids for 4 months. (**F**) Quantitative analysis of mild hyperplasia (white), hyperplasia (grey) and dysplasia (black) in control plasmid (HPV16+pHm6) or HoxA5 treated K14-HPV16 (HPV16+pHoxA5) mice. HoxA5 treated mice showed a significantly higher proportion of mild hyperplasia, with a significantly reduced incidence of hyperplasia and dysplasia compared to control treated HPV16 mice (* p<0.05); n = 15). (**G**) Photomicrograph of Ki-67 positive keratinocytes in ear tissue of 5 month old K14-HPV16 mice following 4 months of treatment with control (HPV16+pHm6) or HoxA5 HPV16+pHoxA5) expression plasmids. (**H**) Quantitation of Ki-67 positive cells in ear tissue of 5 month old K14-HPV16 mice following 4 months of treatment with control (HPV16+pHm6) or HoxA5 HPV16+pHoxA5) expression plasmids (n = 3; ** p<0.05).

We topically applied of 25 μg of HoxA5 expression plasmid (pHoxA5) or control (empty plasmid; pHm6) weekly to ears of 1-month-old K14-HPV16 mice, and mice received 1X/week application until 5-months-of-age. We first confirmed that topical application of 25 μg of HoxA5 expression plasmid resulted in a significant increase in levels of HoxA5 mRNA expression in skin of treated K14-HPV16 mice (Fig 6A). The increased HoxA5 expression was also accompanied by increased TSP-2 mRNA and reduced VEGF-A mRNA (Fig 6A), and was similar in magnitude to changes in gene expression observed following 4 months of activation of the transgene in the HPV16/HoxA5 mice (Fig 5A). CD31 analysis revealed a significantly lower vascular density in 5 month old HPV16 mice treated with topical HoxA5 for 4 months (Fig 6B) and was strikingly similar to the approximately 30% reduction in vascular density observed in the HPV16/HoxA5 transgenic mice at this age (Fig 5C). Also, similar to the 5 month old double transgenic HPV16/HoxA5 mice, mast cell influx was significantly reduced following 4 months of treatment with topical HoxA5 (Fig 6C and 6D). Moreover, progression to dysplasia was similarly retarded by topical application as compared to mice treated with control pHm6 plasmid (Fig 6E and 6F), and again similar to retarded neoplastic progression observed in the double transgenic models (Fig 5F and 5G). The reduced progression to dysplasia was also reflected by a significant reduction in the number of proliferative keratinocytes as assessed by Ki-67 positivity (Fig 6G and 6H). Thus HoxA5 reduced mast cell infiltration, angiogenesis and keratinocyte hyperproliferation resulting in decreased neoplastic progression, regardless of whether HoxA5 was delivered constitutively through expression in ECs or when applied topically to developing neoplastic skin lesions.

## Discussion

Previous studies from our laboratory demonstrated that HoxA5 mRNA is expressed in quiescent vessels, but not in angiogenic vessels associated with invasive ductal carcinoma [8]. We now present evidence that re-expression of HoxA5 in angiogenic vasculature *in vivo* inhibits pathological and neoplastic angiogenesis. Whereas previous studies on HoxA5 *in vivo* employed global loss of function mutants, the present study represents the first inducible, gain-of-function model for a Hox gene with expression restricted to EC. Moreover, we also show that topical application of a HoxA5 transgene is effective in impairing angiogenesis and progression of premalignant dysplasia *in vivo*.

As endogenous HoxA5 is normally expressed in quiescent, mature vessels, we anticipated that sustained ectopic expression of EC HoxA5 would not dramatically influence the function of quiescent or mature vessels. Indeed, we observed that expression of a HoxA5 transgene in ECs starting 3 weeks after birth yielded only a moderate reduction in vascular complexity as reflected by a reduced number of vascular skeletons with no effect on overall vessel density. Mice constitutively expressing HoxA5 also maintained the ability to increase permeability in response to acute stimulation indicating that vascular function was not compromised.

In contrast to the limited impact of HoxA5 during early post-natal vascular development, sustained expression of HoxA5 in mature, adult EC under pathological conditions where the endogenous gene is normally suppressed, resulted in a striking reduction in neoangiogenesis and impaired both wound healing and neoplastic progression.

It is worth noting that angiogenic vasculature in healing wounds is regulated by multifactorial programs involving multiple angiogenic pathways. Whereas administration of anti-VEGF agents can reduce angiogenic sprouting, other angiogenic pathways maintain sufficient levels of neo-angiogenesis allowing wound closure to proceed normally [26,27]. In contrast, as demonstrated herein, sustained HoxA5 significantly inhibits wound angiogenesis and delays wound closure, indicating that HoxA5 may represent a novel, comprehensive approach to managing angiogenesis elicited by both VEGF and non-VEGF-dependent pathways.

In addition to down-regulating VEGFA, HoxA5 enhances expression of the anti-angiogenic TSP-2 which acts to limit wound angiogenesis. Previous studies showed that while deletion of TSP-2 does not impact embryonic vascular development, pathological angiogenesis in response to wounding is enhanced and wound closure is accelerated when TSP-2 is absent [28].

The K14-HPV16 mouse model of squamous carcinogenesis has been extensively used to study both the onset of tumor angiogenesis, as well as its critical role in neoplastic progression [18,29]. B cells and humoral immunity potentiate angiogenic programming of neoplastic tissue in K14-HPV16 mice, resulting in increased mast cell infiltration and subsequent release of pro-angiogenic factors that activate and sustain angiogenesis, and directly support neoplastic progression [17,18,21].^.^ We revealed that endothelial cell expression of HoxA5 during neoplastic progression significantly attenuated angiogenesis and inhibited ongoing progression to the fully dysplastic phenotype, accompanied by reduced VEGFA, increased TSP-2 and a reduced influx of pro-angiogenic mast cells. Moreover, direct topical application of a HoxA5 transgene to neoplastic skin in K14-HPV16 mice resulted in a similar impaired progression to high grade dysplasia accompanied by a similar reduction in angiogenesis, mast cell recruitment and concomitant changes in gene expression. We previously reported that topically applied Hox expression plasmids could be incorporated and expressed by proliferating, angiogenic EC cells in treated areas, and to a lesser extent in proliferating basal keratinocytes, without distal expression [16,30]. Herein, the HoxA5 transgene was also not detected in peripheral blood or distant organs indicating that the topically-applied plasmids remained highly localized to sites of application.

It is possible that incorporation of topically applied HoxA5 into neoplastic cells (keratinocytes) directly influence their behavior. Although we noted a marked decrease in the presence of keratinocytes in areas treated with topical HoxA5, we observed a similar reduction in Ki-67 positivity in HoxA5 transgenic mice where HoxA5 expression is restricted to ECs, indicating that its primary mode of action is in limiting neoplastic progression via inhibition of angiogenesis. Nonetheless, topically applied HoxA5 may directly impact keratinocyte behavior to alter invasive potential [43,44]. Thus future studies are warranted to investigate how HoxA5 might directly alter keratinocyte invasion and SCC development.

Previous studies have also indicated a broad role for HoxA5 during embryonic development. Deletion of both copies of HoxA5 results in an embryonic lethal phenotype, and 50% of heterozygous mutants die at birth or shortly thereafter [31]. Mice lacking HoxA5 presented posterior homeotic transformations of the cervico-thoracic region [31], respiratory tract defects [10], intestinal and stomach differentiation defects [11,32], impaired thyroid gland development [33] and mammary epithelium growth and differentiation defects [34]. Ectopic expression of HoxA5 in neurons during development leads to sensory and motor defects [35]. HoxA5 expression is also an important regulator of hematopoietic lineage determination and maturation, and promotes myeloid differentiation [36,37]. Less is known regarding the role for HoxA5 in adult tissues. Increased breast tumorigenesis via loss of p53 has been linked to loss of HoxA5 via promoter methylation [38]. Other studies noted dysregulated HoxA5 expression in mouse pulmonary EC linked with pulmonary hypertension, but its precise role in EC function was not investigated [39]. HoxA5 expression can be attenuated by mir130a, which is induced by VEGF, as well as inflammatory factors [40] which likely contribute to loss of endogenous HoxA5 in tumor-associated vasculature.

Given the potential range of effects of HoxA5 in adult tissue, targeted expression of HoxA5 to endothelial cells allowed us to examine its role specifically during angiogenesis. The *Tie2-tTA* driver used for this purpose expresses tTA specifically in arteries, veins, and capillaries in all organs including uterus, brain and liver at different levels depending on the organ [12]. Thus, HoxA5 could be repressed during development and then selectively activated in adult EC when endogenous levels are reduced during tumor-induced angiogenesis. Although the TIE2 promoter is also expressed in some hematopoietic stem cells, including a subset of monocytes that can contribute to tumor angiogenesis [41,42], the number of these cells infiltrating developing neoplastic skin in K14-HPV16 mice is low (L. Coussens, unpublished), and thus consistent with undetectable levels of the HoxA5 transgene in peripheral blood or bone marrow cells.

In summary we provide evidence for a novel anti-angiogenic approach to limit neoplastic progression. By restoring expression of HoxA5 to angiogenic vasculature, either using a murine genetic model or via topical application of a HoxA5 transgene, both wound and tumor-induced angiogenesis and neoplastic progression were inhibited.

## Supporting Information

S1 Fig (A) Relative expression of HoxA5 in tissues isolated from 18 day old tTA and HoxA5-tTA mice in which Dox was withdrawn immediately following birth. RNA was isolated from skin, and lung of tTA and HoxA5-tTA mice and relative levels of the HoxA5 transgene mRNA were analyzed by real time PCR (n = 5). (B) Real time PCR analysis of mRNA expression levels for TSP-2 in skin and lung of the same mice used in (A).
(TIF)

S2 Fig Real time PCR analysis of HoxA5 mRNA expression levels in ear tissue harvested 5 month old HoxA5-tTA (HoxA5), K14-HPV16 (HPV16) or K14-HPV16/HoxA5-tTA (HPV16/HoxA5) mice after removal of DOX from the diet for 4 months. (n = 3).
(TIF)

S3 Fig (A) Immunofluorescent staining of the vasculature using CD31 (green) in Oct-embedded frozen tissue sections of 4 month old mice. K14-HPV16/HoxA5 mice (lower panel) exhibit narrower, less tortuous vessels in the underlying dermal area as compared to skin from age-matched K14-HPV16 mice (upper panel). (B) Quantitation of CD31 Immunofluroescent capillaries in dermis from ear tissue of 4 month old K14-HPV16 and HPV16/HoxA5 mice (n = 6). (C) Micrographs of mast cell infiltrate, confirmed by toluidine blue staining, in dermal areas of ear skin of 4 month old K14-HPV16 and HPV16/HoxA5 mice. (D) Quantitation of mast cell infiltrate in ear skin of K14-HPV16 and HPV16/HoxA5 mice (n = 6). (E) Quantitative analysis of mild hyperplasia (white), hyperplasia (grey) and dysplasia (black) in control K14-HPV16 or HPV16/HoxA5 mice. HoxA5-treated mice exhibited a significantly higher proportion of mild hyperplasia, with a significantly reduced incidence of hyperplasia and dysplasia compared to control ear skin from treated K14-HPV16 mice (* p<0.05; n = 8).
(TIF)

S4 Fig Real time PCR analysis of HoxA5 transgene expression levels in 5-month-old K14-HPV16 mice treated with control (HPV16+pHm6) or HoxA5 (HPV16+pHoxA5) expression plasmids for 4 months. RNA was collected from various organs indicated and mRNA levels for HoxA5 assessed (n = 3).
(TIF)
