# Correction: Sustained Endothelial Expression of HoxA5 *In Vivo* Impairs Pathological Angiogenesis And Tumor Progression

**DOI:** 10.1371/journal.pone.0148833

**Published:** 2016-02-03

**Authors:** Ileana Cuevas, Hans Layman, Lisa Coussens, Nancy Boudreau

There is an error in paragraph nine of the Discussion section. The authors incorrectly summarized the findings of the cited paper. The correct sentence is: Previous studies have also indicated a broad role for HoxA5 during embryonic development and 50% of homozygous null HoxA5 mice die at birth or shortly thereafter.
