# Supplementary figures and images for: Sustained Endothelial Expression of HoxA5 In Vivo Impairs Pathological Angiogenesis And Tumor Progression
Source: PLoS One. 2015 Mar 30;10(3):e0121720. doi: 10.1371/journal.pone.0121720 (PMC4379087; doi:10.1371/journal.pone.0121720)

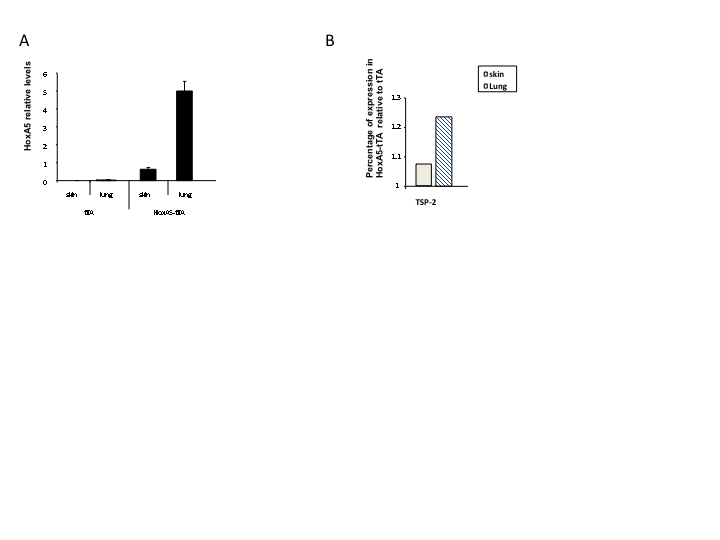

Supplement: S1 Fig — (A) Relative expression of HoxA5 in tissues isolated from 18 day old tTA and HoxA5-tTA mice in which Dox was withdrawn immediately following birth. RNA was isolated from skin, and lung of tTA and HoxA5-tTA mice and relative levels of the HoxA5 transgene mRNA were analyzed by real time PCR (n = 5). (B) Real time PCR analysis of mRNA expression levels for TSP-2 in skin and lung of the same mice used in (A). (TIF) [file pone.0121720.s001.tif]

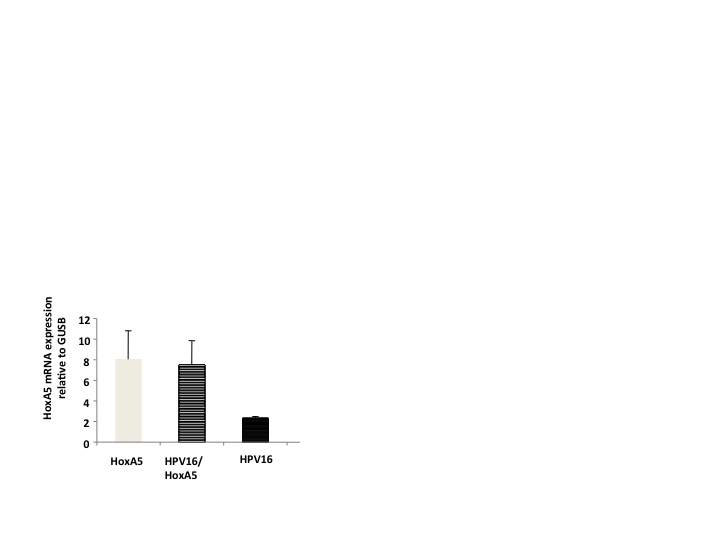

Supplement: S2 Fig — (n = 3). (TIF) [file pone.0121720.s002.tif]

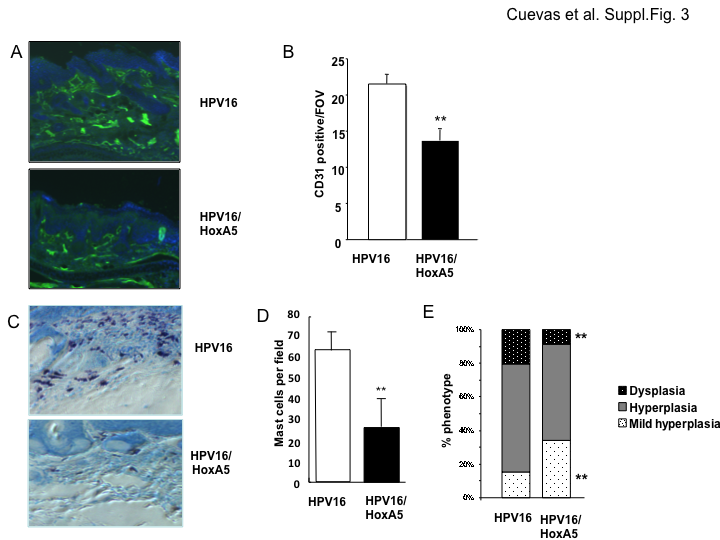

Supplement: S3 Fig — (A) Immunofluorescent staining of the vasculature using CD31 (green) in Oct-embedded frozen tissue sections of 4 month old mice. K14-HPV16/HoxA5 mice (lower panel) exhibit narrower, less tortuous vessels in the underlying dermal area as compared to skin from age-matched K14-HPV16 mice (upper panel). (B) Quantitation of CD31 Immunofluroescent capillaries in dermis from ear tissue of 4 month old K14-HPV16 and HPV16/HoxA5 mice (n = 6). (C) Micrographs of mast cell infiltrate, confirmed by toluidine blue staining, in dermal areas of ear skin of 4 month old K14-HPV16 and HPV16/HoxA5 mice. (D) Quantitation of mast cell infiltrate in ear skin of K14-HPV16 and HPV16/HoxA5 mice (n = 6). (E) Quantitative analysis of mild hyperplasia (white), hyperplasia (grey) and dysplasia (black) in control K14-HPV16 or HPV16/HoxA5 mice. HoxA5-treated mice exhibited a significantly higher proportion of mild hyperplasia, with a significantly reduced incidence of hyperplasia and dysplasia compared to control ear skin from treated K14-HPV16 mice (* p<0.05; n = 8). (TIF) [file pone.0121720.s003.tif]

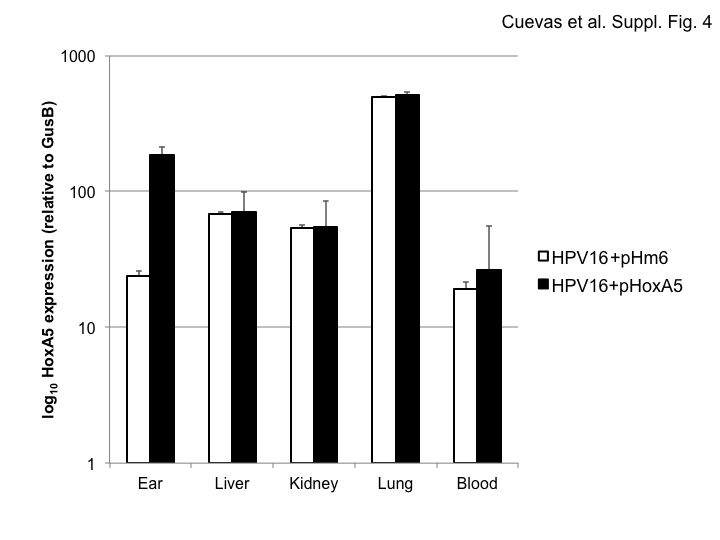

Supplement: S4 Fig — RNA was collected from various organs indicated and mRNA levels for HoxA5 assessed (n = 3). (TIF) [file pone.0121720.s004.tif]
